# Estimation of the hydrogen concentration in rat tissue using an airtight tube following the administration of hydrogen via various routes

**DOI:** 10.1038/srep05485

**Published:** 2014-06-30

**Authors:** Chi Liu, Ryosuke Kurokawa, Masayuki Fujino, Shinichi Hirano, Bunpei Sato, Xiao-Kang Li

**Affiliations:** 1 Division of Transplantation Immunology, National Research Institute for Child Health and Development; 2 AIDS Research Center, National Institute of Infectious Diseases; 3 MiZ Co., Ltd., Kanagawa; Japan; 4 Preclinical Medical Institute, Gannan Medical University, Jiangxi, China; 5 These authors contributed equally to this work.

## Abstract

Hydrogen exerts beneficial effects in disease animal models of ischemia-reperfusion injury as well as inflammatory and neurological disease. Additionally, molecular hydrogen is useful for various novel medical and therapeutic applications in the clinical setting. In the present study, the hydrogen concentration in rat blood and tissue was estimated. Wistar rats were orally administered hydrogen super-rich water (HSRW), intraperitoneal and intravenous administration of hydrogen super-rich saline (HSRS), and inhalation of hydrogen gas. A new method for determining the hydrogen concentration was then applied using high-quality sensor gas chromatography, after which the specimen was prepared via tissue homogenization in airtight tubes. This method allowed for the sensitive and stable determination of the hydrogen concentration. The hydrogen concentration reached a peak at 5 minutes after oral and intraperitoneal administration, compared to 1 minute after intravenous administration. Following inhalation of hydrogen gas, the hydrogen concentration was found to be significantly increased at 30 minutes and maintained the same level thereafter. These results demonstrate that accurately determining the hydrogen concentration in rat blood and organ tissue is very useful and important for the application of various novel medical and therapeutic therapies using molecular hydrogen.

Molecular hydrogen, a potent free radical scavenger, selectively reduces the levels of hydroxyl radical and peroxynitrite, the most cytotoxic reactive oxygen species (ROS), thereby effectively protecting cells^1^. Hydrogen is also beneficial for use in various novel medical therapeutic applications. Recently, hydrogen was reported to be helpful for treating schistosomiasis-associated chronic liver inflammation; these therapeutic properties are ascribed to the scavenging of hydroxyl radical^2^. Furthermore, many studies have reported the use of hydrogen therapy in different diseases, including those involving the nervous, digestive, cardiovascular and respiratory systems. The protective effects of hydrogen have also been confirmed in different animal models, including the ability to limit the infarct volume of the brain, heart, intestines and kidneys by reducing ischemia-reperfusion injury without altering hemodynamic parameters and providing protection against multiple organ damage elicited by generalized inflammation^3^,^4^,^5^,^6^,^7^,^8^,^9^,^10^,^11^,^12^,^13^,^14^,^15^,^16^,^17^. These studies suggest that hydrogen plays a beneficial role in clinical applications in various organs. However, it is difficult to accurately clarify the hydrogen concentrations in animal organs using different administration methods due to the sensitivity of the detectors and issues related to tissue processing. The present study therefore describes a feasible approach for precisely determining the hydrogen concentration in animal tissues. We modified a hydrogen detection method used in conventional gas chromatography in order to develop a more sensitive and accurate system. Using this novel method, we measured the hydrogen concentrations in the tissue following the administration of different concentrations of hydrogen super-rich water (HSRW) and hydrogen super-rich saline (HSRS) at different point times as well as the inhalation of hydrogen gas at different concentrations. Our results demonstrated the establishment of a method for accurately determining the hydrogen concentration in rat blood and organ tissues. Data regarding the hydrogen concentrations *in vivo* may be very useful and important for the application of various novel medical and therapeutic therapies using molecular hydrogen.

## Results

### The concentrations of hydrogen in the blood and tissue were dependent on the dose of HSRW/HSRS administration and hydrogen gas inhalation

**Figure 1:**
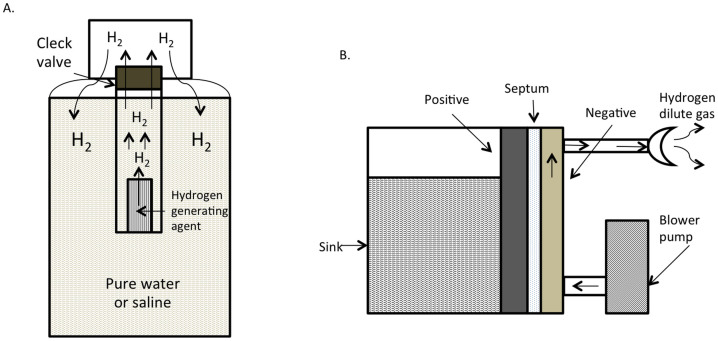
Hydrogen super-rich water, saline and hydrogen gas generation equipment. (A) Water was added to a hydrogen generating agent in the generating apparatus. The inside of the plastic bottle was pressurized through the reaction and hydrogen gas (H_2_) was generated. We produced HSRW or HSRS by stirring the bottle. (B) The hydrogen gas supply system for the biological electrolyte chamber consists of raw water in an electrolyzed chamber, the diaphragm and the electrode plate. Hydrogen gas is directly generated from the electrode plate and cathode, based on the interaction between the fan on the water surface, the cathode gas and the diluted air. The concentration of hydrogen gas near the cathode during electrolysis is always maintained below the lower limit of explosion.

**Figure 2:**
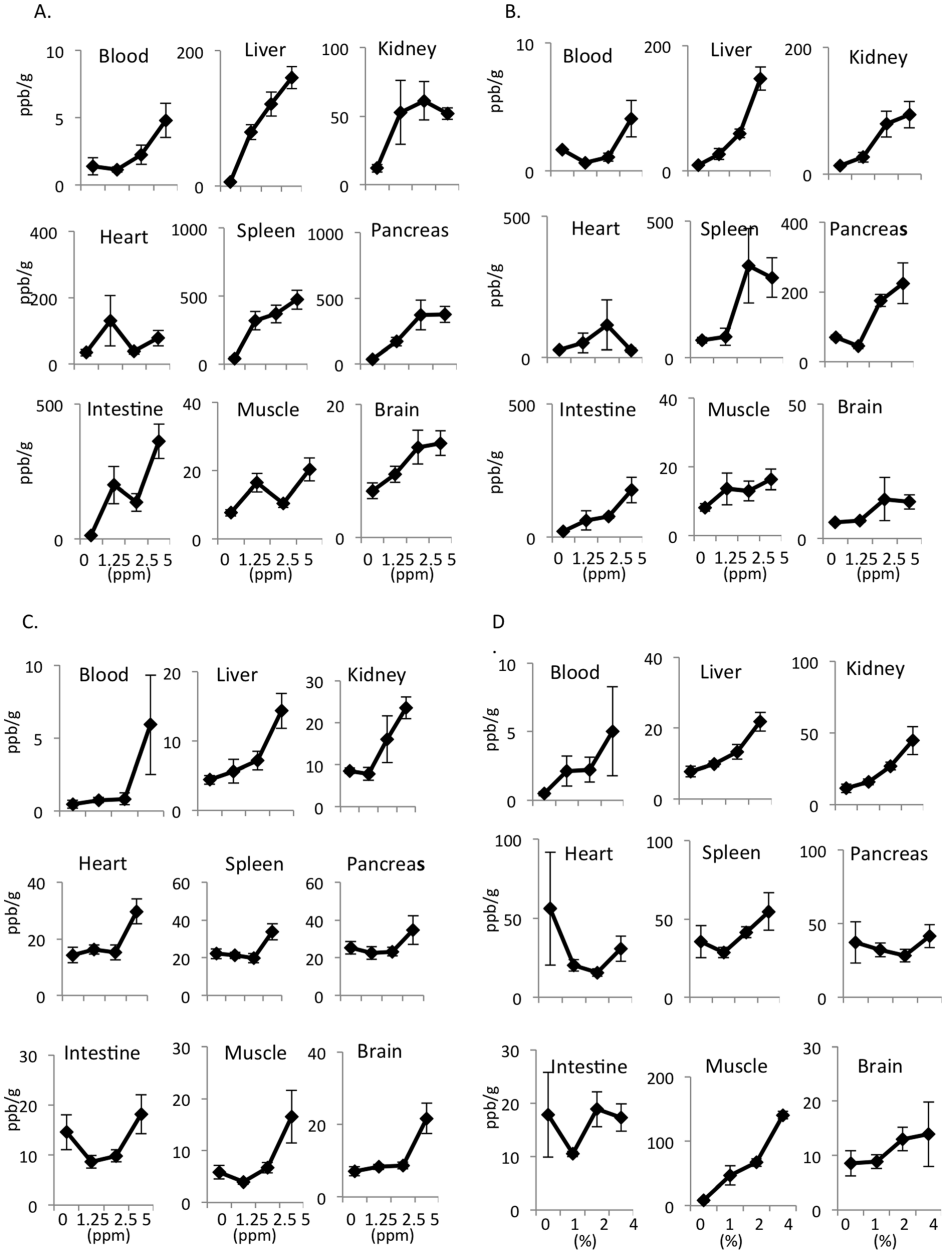
The concentrations of hydrogen in the blood and tissue were dependent on the dose of HSRW/HSRS and hydrogen gas administration. Concentrations of hydrogen in the arterial blood and tissues of the liver, kidneys, heart, spleen, pancreas, intestines, muscles and brain obtained five minutes after (A) the oral administration of HSRW and (B) intraperitoneal administration of HSRS, (C) one minute after the intravenous administration of HSRS and (D) 30 minutes after the inhalation of hydrogen gas. In all experiments, the data were analyzed for more than three rats per group and expressed as the mean ± SEM. The actual number of rats is summarized in Supplemental Table 1.

The hydrogens were prepared as described in the Materials and Methods section (Fig. 1). The hydrogen concentration was measured at five minutes after the oral and intraperitoneal administration of hydrogen at different concentrations (1.25, 2.5 and 5.0 ppm). The hydrogen concentrations in the blood and tissues of the liver, spleen, pancreas and brain exhibited a dose-dependent increase in association with an increase in the concentration of orally administered HSRW (Fig. 2A). The intraperitoneal injection of HSRS also resulted in a dose-dependent increase in the tissue hydrogen concentration (Fig. 2B). Furthermore, the intravenous injection of HSRS induced a dose-dependent increase in the hydrogen concentration (Fig. 2C). Moreover, the hydrogen concentrations in the blood and tissues of the liver, kidneys and spleen increased following the inhalation of hydrogen gas, depending on the dose of hydrogen gas applied (Fig. 2D). In order to assess the efficacy of the current method for determining the hydrogen concentration using a hermetic (airtight) tube under leaky conditions, we performed an additional experiment comparing the differences obtained with and without the application of a cap of gentleMACS tube versus with and without hermetic conditions. As shown in Supplementary Figure 1, the hydrogen concentrations obtained under the hermetic conditions were 70% to 95% lower than those obtained with the current method under hermetic conditions, a significant difference.

### The concentrations of hydrogen in the blood and tissue were dependent on the time after HSRW/HSRS administration and hydrogen gas inhalation

**Figure 3:**
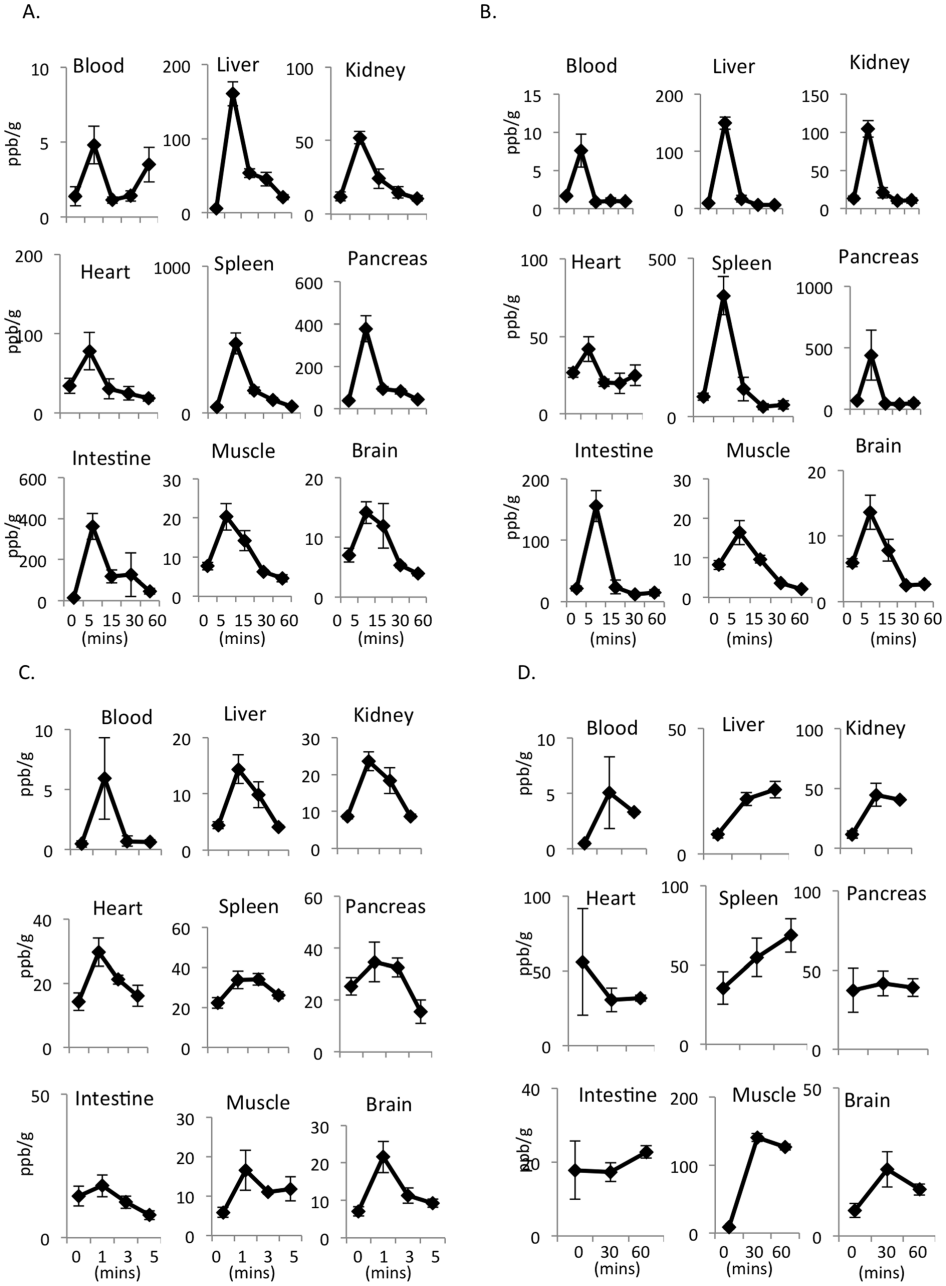
The concentrations of hydrogen in the blood and tissue were dependent on the time after HSRW/HSRS and hydrogen gas administration. Concentrations of hydrogen in the arterial blood and tissues of the liver, kidneys, heart, spleen, pancreas, intestines, muscles and brain obtained at different time points after (A) the oral administration of 5 ppm of HSRW, (B) intraperitoneal administration of 5 ppm of HSRS, (C) intravenous administration of 5 ppm of HSRS and (D) inhalation of 4% hydrogen gas. In all experiments, the data were analyzed for more than three rats per group and expressed as the mean ± SEM. The actual number of rats is summarized in Supplemental Table 2.

The hydrogen concentrations in the blood and tissues were determined 5, 15, 30 and 60 minutes after oral and intraperitoneal administration and 1, 3 and 5 minutes after intravenous administration of 5 ppm of hydrogen. All values reached a maximum at five minutes after the oral and intraperitoneal administration of HSRW (Fig. 3A) and HSRS (Fig. 3B), compared to one minute for the intravenous administration of HSRS. The concentrations of hydrogen in the blood and tissues then slowly decreased (Fig. 3A, B, C). The decline in the hydrogen concentrations in the blood and tissues observed after reaching the highest level at five minutes was more rapid following intraperitoneal administration (Fig. 3B) than oral administration (Fig. 3A). In contrast, the concentrations of hydrogen peaked at one minute after intravenous treatment, then gradually decreased from one to five minutes (Fig. 3C). The inhalation of hydrogen gas resulted in slower elevation of the hydrogen concentration than that achieved with intraperitoneal, intravenous or oral administration. However, the elevated hydrogen concentrations were maintained for at least 60 minutes after inhalation (Fig. 3D).

**Figure 4:**
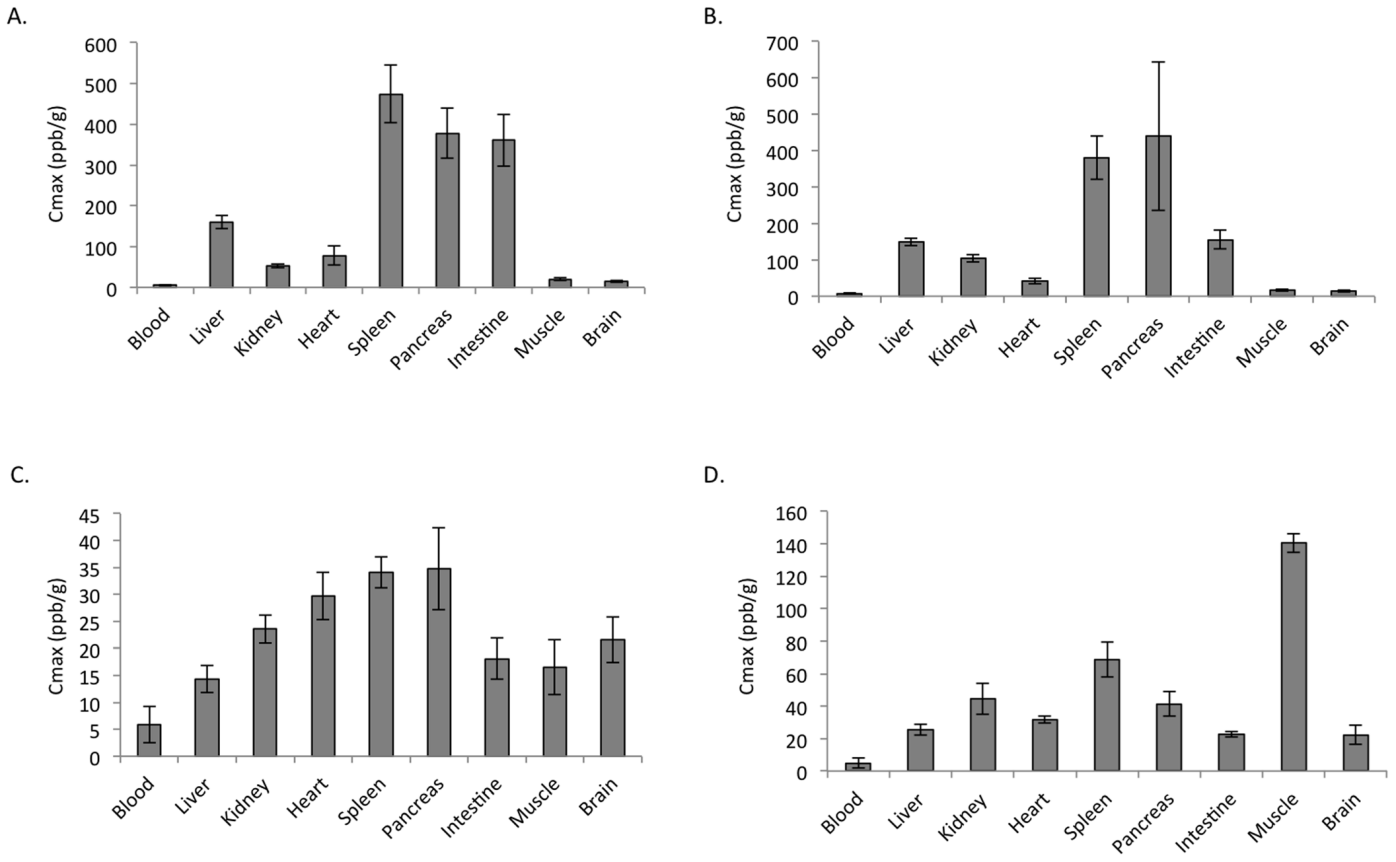
Cmax analysis of the hydrogen concentrations in different tissues. Cmax (peak) of the hydrogen concentrations in the arterial blood and tissues of the liver, kidneys, heart, spleen, pancreas, intestines, muscles and brain after (A) the oral administration of 5 ppm of HSRW, (B) intraperitoneal administration of 5 ppm of HSRS, (C) intravenous administration of 5 ppm of HSRS and (D) inhalation of 4% hydrogen gas. In all experiments, the data were analyzed for more than three rats per group and expressed as the mean ± SEM. The actual number of rats is summarized in Supplemental Table 2.

Furthermore, a Cmax analysis showed that the oral administration of HSRW resulted in a high hydrogen concentration (more than 300 ppb/g) in the tissues of the spleen, small intestine and pancreas (Fig. 4A). Meanwhile, the intraperitoneal administration of HSRS resulted in a high hydrogen concentration in the spleen and pancreas (more than 300 ppb/g) (Fig. 4B). However, the hydrogen concentrations were not high (more than 35 ppb/g) in any of the tissues after intravenous treatment (Fig. 4C). Interestingly, the inhalation of hydrogen gas resulted in the highest hydrogen concentration in the muscle (more than 140 ppb/g; Fig. 4D).

**Figure 5:**
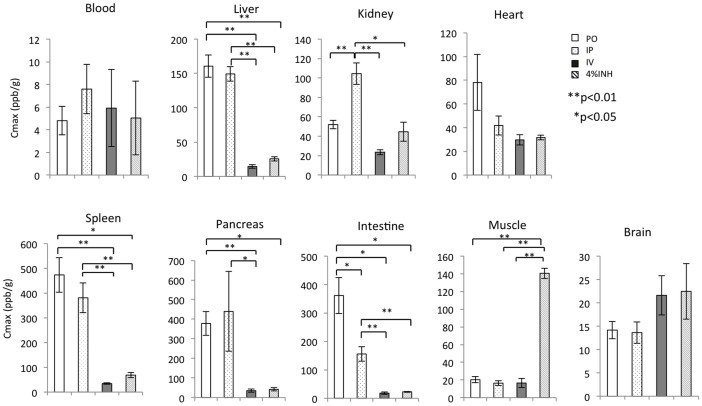
Cmax analysis of the hydrogen concentrations using different administration methods. Comparison of the Cmax (peak) of the hydrogen concentrations in the arterial blood and tissues of the liver, kidneys, heart, spleen, pancreas, intestines, muscles and brain after the oral, intraperitoneal, intravenous administration of 5 ppm of HSRW/HSRS and 4% hydrogen gas inhalation. In all experiments, the data were analyzed for more than three rats per group and expressed as the mean ± SEM (*p< 0.05; **p<0.01). The actual number of rats is summarized in Supplemental Tables 1 and 2.

As shown in Figure 5, we compared the peak values of the hydrogen concentration after the various forms of administration (oral, intraperitoneal, intravenous and inhalation). The hydrogen concentrations in the liver, kidneys, spleen, pancreas and intestines were significantly higher after oral and intraperitoneal treatment than those observed following intravenous or inhalation administration. In contrast, the inhalation of hydrogen gas induced significantly higher hydrogen concentrations in the muscle and slightly higher hydrogen concentrations in the brain compared to the other modes of administration.

## Discussion

In this study, we developed a modified method to detect the hydrogen concentration in rat tissue. There are two advantages to this modified method. The first advantage is the utilization of hermetic airtight conditions using gentleMACS, which was shown to prevent the leakage of hydrogen from the sampling tissue during processing. As shown in Supplementary Figure 1, the hydrogen concentrations in the tissues were significantly lower under the hermetic airtight conditions than those obtained using the traditional conditions, thus minimizing hydrogen leakage from the tissue samples during processing.

The second advantage is the application of high-quality sensor gas chromatography, SGHA-P1. SGHA-P1 is equipped with a semiconductor gas sensor. Compared with conventional gas chromatography, which employs a thermal conductivity detector, SGHA-P1 can be used to detect a hydrogen concentration of 10 ppb, a value that is approximately 10 to 100 times higher than the limit of detection of conventional gas chromatography, the ion-selective electrode and platinum catalyzed methylene blue method. Using the modified method, we found that the hydrogen concentrations in the rat blood and tissues peaked at five minutes after oral and intraperitoneal administration. In contrast, the hydrogen concentrations reached a peak at one minute after intravenous administration, then gradually decreased from one to five minutes, finally reaching the control level. The hydrogen concentrations in the rat blood and tissues reached their highest levels at 30 minutes after the inhalation of hydrogen gas, then began to exhibit a slow downward trend from 30 to 60 minutes. These results indicate that we successfully established a method for determining the hydrogen concentration that can be applied to stably detect hydrogen in various tissues after treatment with HSRW/HSRS and hydrogen gas. To date, many researchers have reported the hydrogen concentrations *in vivo* following exogenous hydrogen treatment using rodent models^1^,^9^,^11^,^18^,^19^,^20^; however, there are no previous reports comparing the hydrogen concentrations in multiples tissues using several routes of administration, doses of hydrogen and time points after administration.

Recent evidence indicates that HSRW, HSRS and inhaled hydrogen gas have antioxidant and anti-apoptotic properties that can protect organs from ischemia-reperfusion injury by selectively scavenging detrimental ROS^1^. The mechanism of action of hydrogen in this model involves the ability of this compound to prevent oxidative damage, as indicated by decreased nucleic acid oxidation and lipid peroxidation^1^,^21^. Furthermore, many studies have provided evidence that hydrogen plays a protective role against acute and chronic injury in various organs^3^,^4^,^5^,^6^,^7^,^8^,^9^,^10^,^11^,^12^,^13^,^14^,^15^,^16^,^17^. According to the present data, the hydrogen concentrations in the animal tissues reached a maximum five minutes after oral and intraperitoneal treatment; therefore, the best time point for treatment with hydrogen, with respect to obtaining significant ROS scavenging effects, may be five minutes before the start of experiment.

Inflammatory processes are known to be closely linked with oxidative stress, and several studies have addressed the potential of hydrogen for use in anti-inflammatory therapy. In animal models of inflammatory disorders, it has been reported that hydrogen attenuates inflammation in the setting of hepatitis, colitis, pancreatitis, obstructive jaundice and sepsis^13^,^22^,^23^. Under inflammatory conditions, hydrogen treatment has been found to significantly reduce the levels of interleukin (IL)-6 and tumor necrosis factor (TNF)-α, as well as other inflammation-associated molecules, including IL-12 and interferon (IFN)-γ^4^,^6^,^11^. The present results also showed high concentrations of hydrogen in the rat spleen, small intestine, pancreas, liver and kidneys after oral and intraperitoneal treatment and in the muscles, lungs and testis (preliminary results) after the inhalation of hydrogen gas. Based on these data, clinicians and researchers can choose the more efficient route of treatment and explore whether hydrogen inhibits inflammatory diseases in these organs.

Hydrogen has not been reported to be toxic at effective doses, and overdoses are unlikely, as excess hydrogen is expired via the lungs^16^. This phenomenon contrasts with that of antioxidants, such as vitamins C and E, for which the effective dose in humans is higher than the upper limit of tolerated intake^13^,^17^,^23^. According to the present results and previous findings, there is a significant possibility that chronic diseases caused by ROS can be prevented with treatment with daily oral or inhaled hydrogen. In addition, these treatments may increase muscle strength and improve infertility. However, the efficient concentrations of hydrogen required to treat acute and chronic inflammatory diseases have not been clarified. The novel method for estimating the hydrogen concentration *in vivo* described in this study may help to determine the cut-off values for this parameter, at least in various experimental animal models. The hydrogen present in organ tissue easily diffuses into the air following trimming and homogenization. Therefore, it is difficult to accurately estimate the concentration of hydrogen in organ tissue, and these values are influenced by the background to a certain extent. However, our novel method demonstrated almost no background effects.

Many experiments have demonstrated the therapeutic effects of hydrogen based on its anti-inflammatory mechanism of action. However, there are no previous reports showing what hydrogen concentration in organ tissue is necessary to achieve the therapeutic benefits of hydrogen. In order to assess the correlation between the hydrogen concentration and its biological effects, we performed experiments involving treatment for kidney ischemia-reperfusion injury with supersaturated hydrogen super-rich saline (HSRS). In this experiment, we used two concentrations of HSRS (1.6 ppm and 5 ppm) in mice. Consequently, the survival times and clinical scores significantly improved, with the extent of improvement being dependent on the concentration of HSRS. These results will be described in detail elsewhere (manuscript in preparation). These findings and the findings of the current study clearly demonstrate a correlation between the hydrogen concentration *in vivo* and a clinical improvement. Moreover, the mechanism of acute and chronic inflammation underlying the pathogenesis of disease depends on the degree of local cellular infiltration, as well as infiltration throughout the body, or at least in some lymphoid tissues. Therefore, it is important to know the hydrogen concentration in each organ. The ability to estimate the hydrogen concentration *in vivo*, as demonstrated in the present study, may provide useful information for a precise investigation of the anti-inflammatory effects of hydrogen.

In conclusion, we determined the hydrogen concentrations in various organs using our newly developed method. The results of our analysis may contribute to the use of hydrogen in numerous clinical applications and provide a favorable background for the development of novel clinical therapies.

## Methods

### Animals

Male Wistar rats 8 to 12 weeks of age weighing 180 ~ 200 g were purchased from Shizuoka Laboratory Animal Center (Shizuoka, Japan). All rats were maintained under standard conditions and fed rodent food and water in accordance with the guidelines of the Animal Use and Care Committee of the National Research Institute for Child Health and Development, Tokyo, Japan. All animal manipulations were performed according to the recommendations of the Committee of the Care and Use of Laboratory Animals at the National Research Institute for Child Health and Development, Japan.

### Preparation of HSRW, HSRS and hydrogen gas

HSRW and HSRS were prepared using Hydrogen water 7.0 (ECOMO International Co. Ltd., Fukuoka, Japan) with purified water and saline, respectively (Fig. 1A). The concentration of dissolved hydrogen was 1.25, 2.5 and 5.0 ppm in HSRW and HSRS, as measured using a dissolved hydrogen reagent methylene blue kit (MiZ Co. Ltd, Kanagawa, Japan). Three concentrations (1%, 2% and 4%) of hydrogen gas were prepared using the hydrogen gas supply device (MiZ Co. Ltd.) (Fig. 1B) and measured with the high concentration detector XP-3140 (New Cosmos Electric Co. Ltd., Osaka, Japan).

### HSRW and HSRS treatment protocol

The rats were caged in two groups. The HSRW and HSRS groups were orally administered HSRW and intraperitoneally or intravenously injected with HSRS at three concentrations (1.25, 2.5 and 5.0 ppm), respectively, while the control groups were given distilled water and saline. Arterial blood and tissues of the liver, kidneys, heart, spleen, pancreas, intestines, muscles and brain were obtained at 5, 15, 30 and 60 minutes after the oral and intraperitoneal administration of HSRW and HSRS and at 1, 3 and 5 minutes after the intravenous injection of HSRS.

### Hydrogen gas treatment protocol

The rats were treated with three concentrations of mixed gas (1% hydrogen and 99% air, 2% hydrogen and 98% air and 4% hydrogen and 96% air, respectively) through the hydrogen gas supply device. The hydrogen concentration in the mix gas was determined using the gas detector XP-3140 (New-cosmos Ltd. Co, Tokyo, Japan). The arterial blood and tissues of the liver, kidneys, heart, spleen, pancreas, intestines, muscles and brain were obtained at 30 and 60 minutes after the inhalation of different concentrations of hydrogen gas.

### Tissue dissociation and the determination of the hydrogen concentrations in the arterial blood and tissues

The tissues were placed into gentleMACS tubes (Miltenyi Biotec, Bergisch Gladbach, Germen) filled with pure air (Taiyo Co. Ltd., Tokyo, Japan) immediately after sampling and then homogenized using the gentleMACS^TM^ Octo Dissociator (Miltenyi Biotec). Then, 2 ml of gas in the tube was collected through the rubber layer with a syringe. The hydrogen concentrations were subsequently measured using high-quality Sensor Gas Chromatography (SGHA-P1; FIS Co. Ltd., Hyogo, Japan). Each arterial blood sample was placed in a glass tube filled with the pure air, and 2 ml of gas was collected for measurement. The hydrogen concentration in each blood or tissue sample was defined as follows: Hydrogen concentration (ppb/g; V/V) = A/B × C; where A is the measurement value obtained using the Sensor Gas Chromatograph, B is the blood or tissue weight (g) and C is the volume of the gentleMACS tube (24 ml).

### Statistical analysis

All experiment data are representative of more than three independent experiments, expressed as the mean ± standard error of the mean (SEM) for each experiment. Student's *t*-test was used to compare the paired and unpaired variables.

## Author Contributions

C.L. and R.K. performed the experiment. M.F. and X-K.L. wrote the manuscript. M.F., S.H., B.S. and X.-K.L. designed the experiment and analyzed the data.

## Supplementary Material

Supplementary Information supplementary informations
