# Supplementary material for: Estimation of the hydrogen concentration in rat tissue using an airtight tube following the administration of hydrogen via various routes
Source: Sci Rep. 2014 Jun 30;4:5485. doi: 10.1038/srep05485 (PMC4074787; doi:10.1038/srep05485)
Supplement: Supplementary Information — s [file srep05485-s1.pdf]

Supplementary Information

Title:
Estimation of the hydrogen concentration in rat tissue using an airtight tube following the administration of hydrogen via various routes

Authors:
Chi Liu, Ryosuke Kurokawa, Masayuki Fujino, Shinichi Hirano, Bunpei Sato, and Xiao-Kang Li

Supplementary Figure1

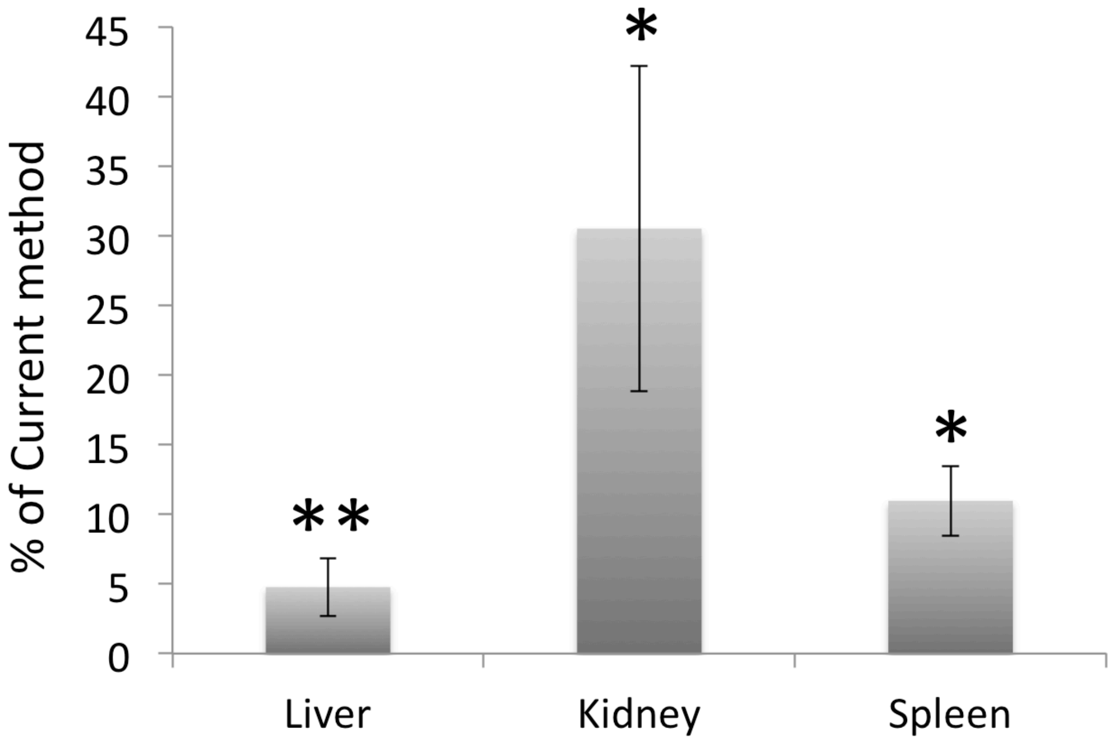

Supplementary Table 1

HSRW/OP/5min	0ppm	1.25ppm	2.5ppm	5ppm
Blood	4	6	6	12
Liver	4	6	6	12
Kidney	4	6	6	12
Heart	4	6	6	12
Spleen	4	6	6	12
Pancreas	4	6	6	12
Intestine	4	6	6	12
Muscle	4	6	6	9
Brain	4	6	6	9

HSRS/IP/5min	0ppm	1.25ppm	2.5ppm	5ppm
Blood	3	3	3	6
Liver	3	3	3	6
Kidney	3	3	3	6
Heart	3	3	3	6
Spleen	3	3	3	6
Pancreas	3	3	3	6
Intestine	3	3	3	6
Muscle	3	3	3	3
Brain	3	3	3	3

HSRS/IV/1min	0ppm	1.25ppm	2.5ppm	5ppm
Blood	5	3	3	9
Liver	5	3	3	9
Kidney	5	3	3	9
Heart	5	3	3	9
Spleen	5	3	3	9
Pancreas	5	3	3	9
Intestine	5	3	3	9
Muscle	5	3	3	9
Brain	5	3	3	9

Hydrogen gas/30min	0%	1%	2%	4%
Blood	5	3	6	3
Liver	5	3	6	3
Kidney	5	3	6	3
Heart	5	3	6	3
Spleen	5	3	6	3
Pancreas	5	3	6	3
Intestine	5	3	6	3
Muscle	5	3	6	3
Brain	5	3	6	3

Supplementary Table 2

HSRW/OP/5ppm	0min	5min	15min	30min	60min
Blood	4	12	9	9	9
Liver	4	12	9	9	9
Kidney	4	12	9	9	9
Heart	4	12	9	9	9
Spleen	4	12	9	9	9
Pancreas	4	12	9	9	9
Intestine	4	12	9	9	9
Muscle	4	9	9	6	6
Brain	4	9	9	6	6

HSRS/IP/5ppm	0min	5min	15min	30min	60min
Blood	3	6	3	6	6
Liver	3	6	3	6	6
Kidney	3	6	3	6	6
Heart	3	6	3	6	6
Spleen	3	6	3	6	6
Pancreas	3	6	3	6	6
Intestine	3	6	3	6	6
Muscle	3	3	3	3	3
Brain	3	3	3	3	3

HSRS/IV/5ppm	0min	1min	3min	5min
Blood	5	9	5	3
Liver	5	9	5	3
Kidney	5	9	5	3
Heart	5	9	5	3
Spleen	5	9	5	3
Pancreas	5	9	5	3
Intestine	5	9	5	3
Muscle	5	9	5	3
Brain	5	9	5	3

Hydrogen gas/4%	0min	30min	60min
Blood	5	3	3
Liver	5	3	3
Kidney	5	3	3
Heart	5	3	3
Spleen	5	3	3
Pancreas	5	3	3
Intestine	5	3	3
Muscle	5	3	3
Brain	5	3	3

Supplementary figure legends

Supplementary Figure 1: Comparison of the hydrogen concentration in tissues after oral administration of HSRW with and without hermetic condition. The concentration of hydrogen levels in tissues of liver, kidney and spleen at 5min after oral administration of 5 ppm HSRW was determined with and without cap of airtight tube. All experiment data were expressed % of hydrogen concentration of the current method using airtight tube with cap. Data were expressed as the mean ± SEM (n=4). *P < 0.05 and **P < 0.005 vs. value in current method.

Supplementary table legends

Table 1: the number of rat in results that demonstrated that the concentration of hydrogen levels in the blood and tissue was dose-dependent on the HSRW/HSRS and hydrogen gas administration.

Table 2: the number of rat in results that demonstrated that the concentration of hydrogen levels in the blood and tissue was time-dependent after the HSRW/HSRS and hydrogen gas administration.
